# Sex differences in the anatomy of the anterior–superior acetabular rim in relation to pincer-type femoroacetabular impingement in Koreans: a three-dimensional quantitative analysis

**DOI:** 10.1186/s13018-023-03526-w

**Published:** 2023-03-02

**Authors:** Byung Woo Cho, Hyuck Min Kwon, Jun Young Park, Kwan Kyu Park, Taehyun Kim, Woo-Suk Lee

**Affiliations:** 1 Department of Orthopaedic Surgery, Gangnam Severance Hospital, Yonsei University College of Medicine, 211 Eonju-Ro, Gangnam-Gu, Seoul, Republic of Korea; 2 Department of Orthopaedic Surgery, Severance Hospital, Yonsei University College of Medicine, 50-1 Yonsei-Ro, Seodaemun-Gu, Seoul, Republic of Korea; 3 Department of Orthopaedic Surgery, Yongin Severance Hospital, Yonsei University College of Medicine, Yongin, Gyeonggi-do Republic of Korea

## Abstract

**Purpose:**

The aim of this study was to compare the anatomical structures of the acetabular rim around the anterior inferior iliac spine (AIIS) ridge that indicate anterior focal coverage of acetabulum between the sexes using a three-dimensional (3D) model.

**Methods:**

3D models of 71 adults (38 men and 33 women) with normal hip joints were used. Based on the location of the inflection point (IP) of the acetabular rim around the AIIS ridge, the patients were classified into anterior and posterior types, and the ratios thereof for each sex were compared. Coordinates for the IP, the most anterior point (MAP), and the most lateral point (MLP) were obtained and compared between the sexes and between anterior and posterior types.

**Results:**

Coordinates for IPs in men were located anterior and inferior to those in women. MAP coordinates for men were located inferior to those for women, and MLP coordinates for men were located lateral and inferior to those for women. Comparing AIIS ridge types, we noted that coordinates for IPs of the anterior type were located medial, anterior, and inferior to those of the posterior type. Meanwhile, MAP coordinates of the anterior type were located inferior to those of the posterior type, and MLP coordinates of the anterior type were located lateral and inferior to those of the posterior type.

**Conclusion:**

Anterior focal coverage of the acetabulum appears to differ between the sexes, and this difference may affect the development of pincer-type femoroacetabular impingement (FAI). Additionally, we found that anterior focal coverage differs according to anterior or posterior positioning of the bony prominence around the AIIS ridge, which may affect development of FAI.

## Introduction

Pincer-type femoroacetabular impingement (FAI) is caused by global or focal acetabular overcoverage [3, 16], wherein bony contact occurs mainly between osseous deformity of the anterior–superior quadrant of the acetabular rim and the femoral neck. The morphology of the anterior–superior acetabular rim is not a simple curve, and it shows an inflection near the anterior inferior iliac spine (AIIS) ridge, thereby achieving bony prominence [14]. Several studies have reported that this structure can contribute to hip impingement. Hetsroni et al. classified the morphology of the AIIS into three types and reported that hip range of motion (ROM) was different for each type [11]. In their simulation, actual impingements occurred at the bony prominence of the AIIS ridge. Carton et al. distinguished AIIS impingement from subspine impingement, and the intracapsular subspine region indicated the bony prominence of the AIIS ridge [4]. In simulation studies using three-dimensional (3D) models, pincer-type impingement appears to occur around the AIIS ridge [2, 20]. As such, the anterior–superior acetabular rim around the AIIS ridge, including the bony prominence, is considered a cause of hip impingement. However, the features of this anatomical structure are still unclear.

Since there are anatomical differences in the pelvis and acetabulum according to sex [23, 27–29], it can be inferred that there may be differences in the structure of the anterior–superior acetabular rim. In a cadaveric study by Karn et al., men and women were compared by measuring the length of the anterior/posterior acetabular rim based on the location of the AIIS ridge; however, it was difficult to determine the relative position of the AIIS ridge because the acetabular size itself differed between the sexes [14]. Therefore, the purpose of this study was to compare anatomical differences in the anterior–superior acetabular rim between sexes quantitatively by evaluating the bony prominence of the AIIS ridge using a 3D model. We hypothesized that the morphology of AIIS ridges would differ between the sexes.

## Materials and methods

### Patient recruitment

This retrospective computed tomography (CT) scan image analysis study was approved by the institutional review board. Adults aged 20–50 years with normal hip joints who underwent full-length lower extremity CT (CT lower extremity, CT lower extremity angiography, and CT deep vein thrombosis) at our institution between June 2020 and May 2021 were screened. The exclusion criteria were as follows: (1) patients with osteoarthritis or osteophytes of the hip joint; (2) patients with previous hip surgery, hip dysplasia, congenital anomalies, or traumatic deformities; and (3) patients whose pelvis and femurs were not included in the image. Total 143 subjects were screened, and ultimately, 71 subjects (38 men and 33 women) and 142 hips were enrolled in the study.

### 3D model reconstruction and definitions of measurements

**Fig. 1:**
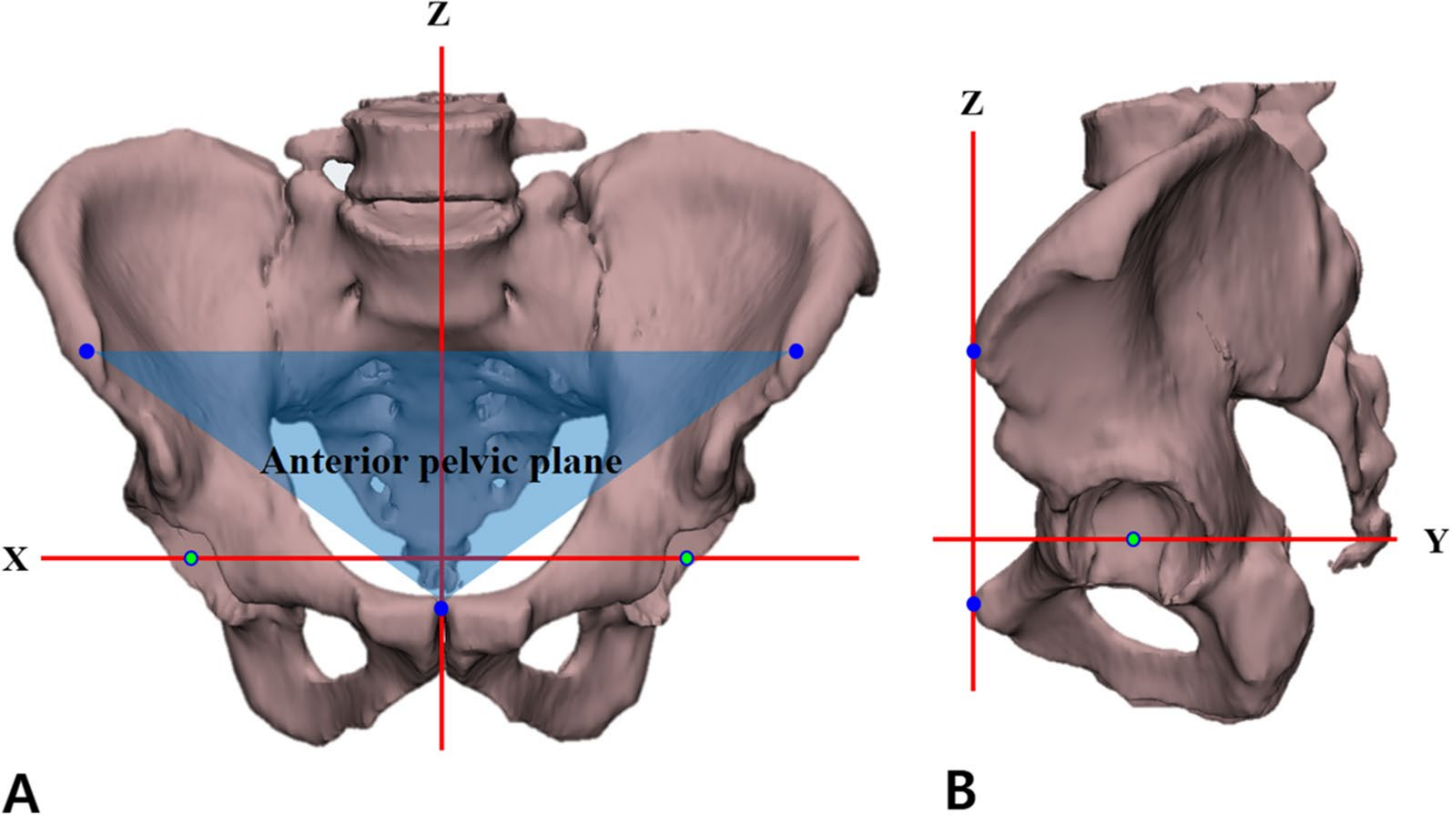
Spatial orientation of X-, Y- and Z-axes.** A** Anterior view.** B** Lateral view

After extracting the patient’s CT scan data as a digital imaging and communications in medicine (DICOM) file, 3D model reconstruction and simulation were performed using Mimics and 3-Matic software (Materialize, Leuven, Belgium). The spatial orientation was as follows: X-axis, a line between both femoral head centers; Z-axis, a line perpendicular to the X-axis that included in the anterior pelvic plane (APP); and Y-axis, a line perpendicular to the X- and Z-axes (Fig. 1). A posture in which the femur’s mechanical axes were parallel to the Z-axis and perpendicular to the X-axis was defined as the default posture.

**Fig. 2:**
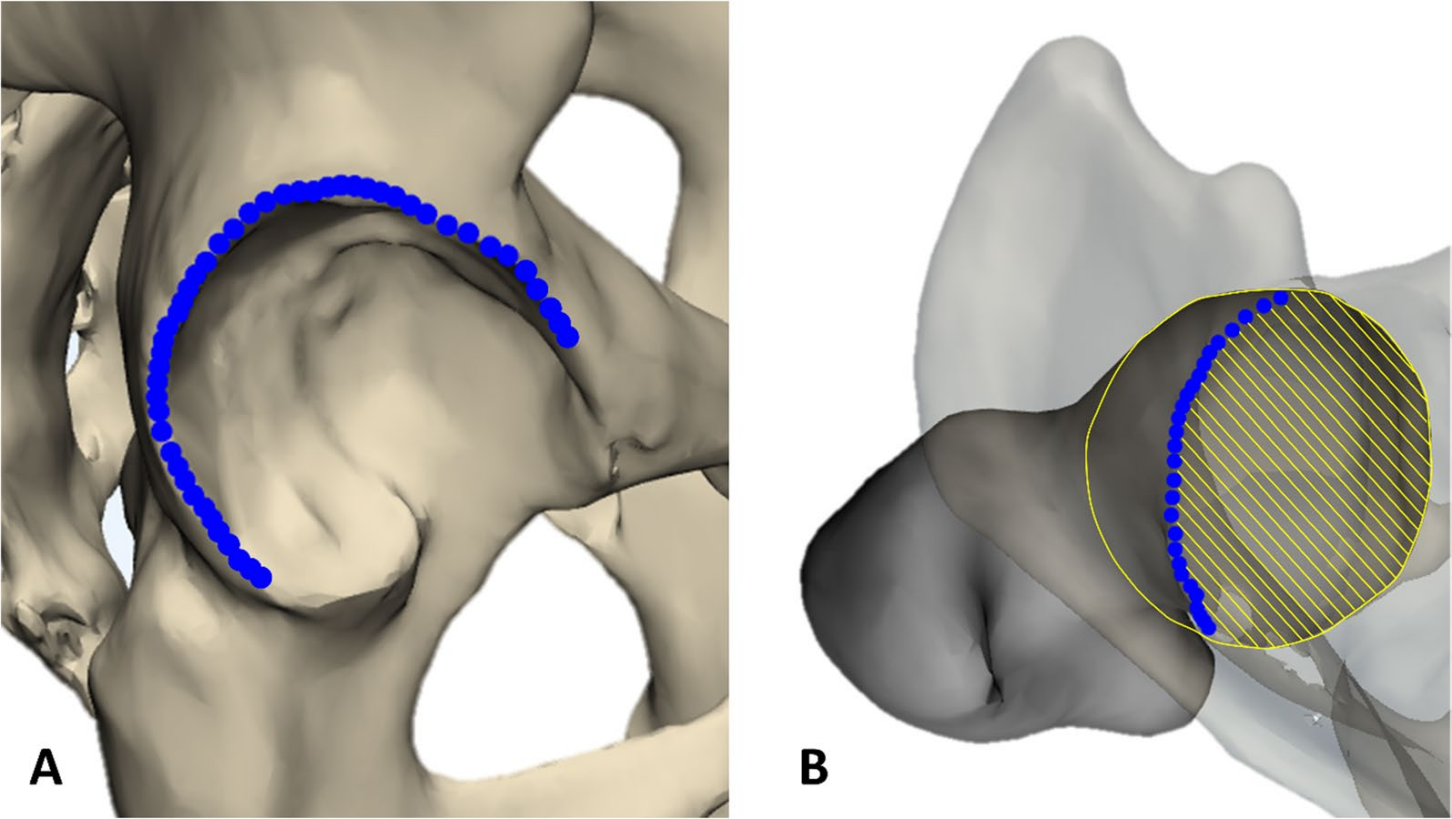
**A** Acetabular rim of the right hip. **B** Measurement of acetabular coverage to the femoral head (hatched area)

The acetabular rim was defined as the connection of the most protruding points (Fig. 2A). Then, the acetabular coverage area of the femoral head in the horizontal plane was calculated. The covered area was defined by using the acetabular rim, and the boundary of the femoral head was set on the basis of the superior hemisphere: Coverage = area covered by acetabulum/area of the femoral head (Fig. 2B).

**Fig. 3.**
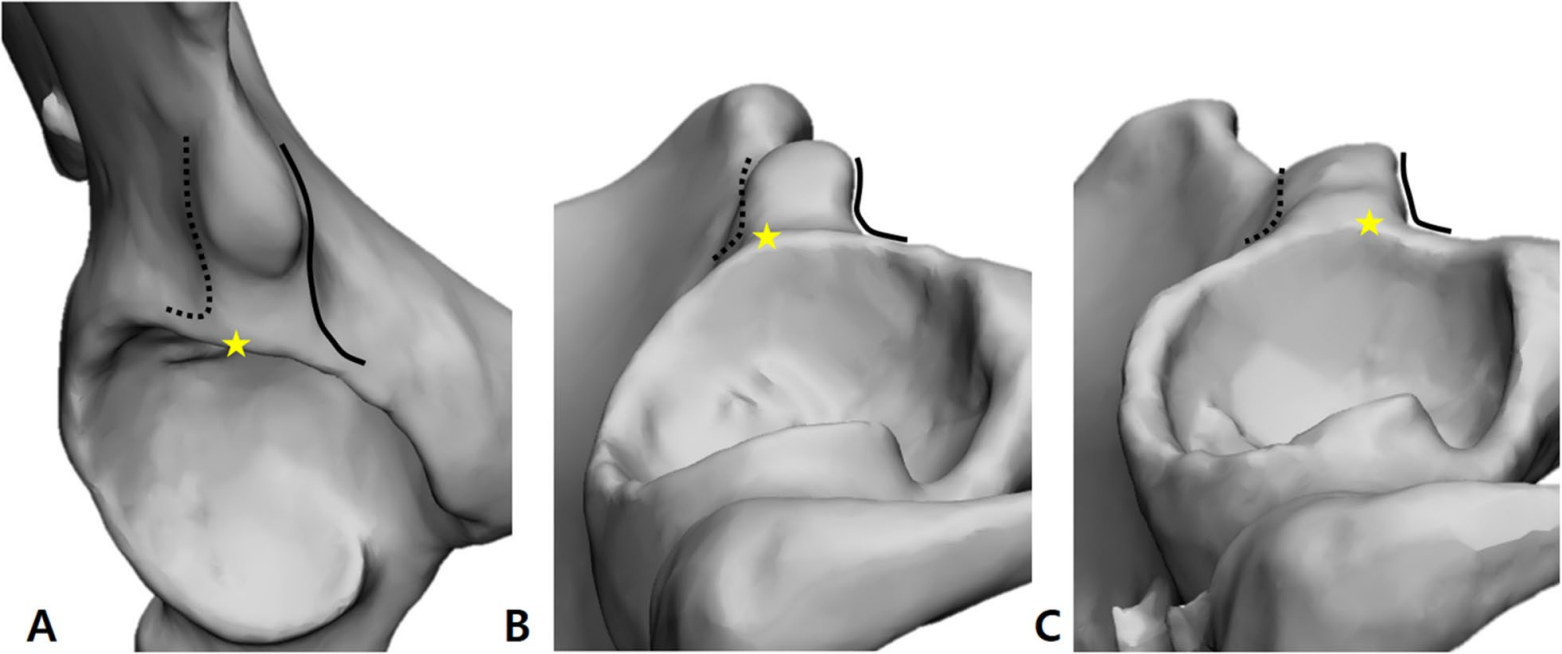
3D models of the right acetabulum. **A** The anterior and posterior edges of the AIIS are identified at the anterior–superior acetabular rim. **B** Example of the posterior type wherein the inflection point is located on the posterior AIIS edge. **C** Example of the anterior type wherein the inflection point is located on the anterior AIIS edge (solid line, anterior AIIS edge; dotted line, posterior AIIS edge; and asterisk, inflection point of acetabular rim)

In all subjects, inflection of the acetabular rim around the AIIS ridge was distinguished, and it was defined as the inflection point (IP) (Fig. 3). After identifying the anterior and posterior edges of the AIIS at the connected portion of the AIIS and acetabular rim, the AIIS ridge shapes were classified into two types (anterior and posterior type) according to which side the IP was closest (Fig. 3B, C).

**Fig. 4:**
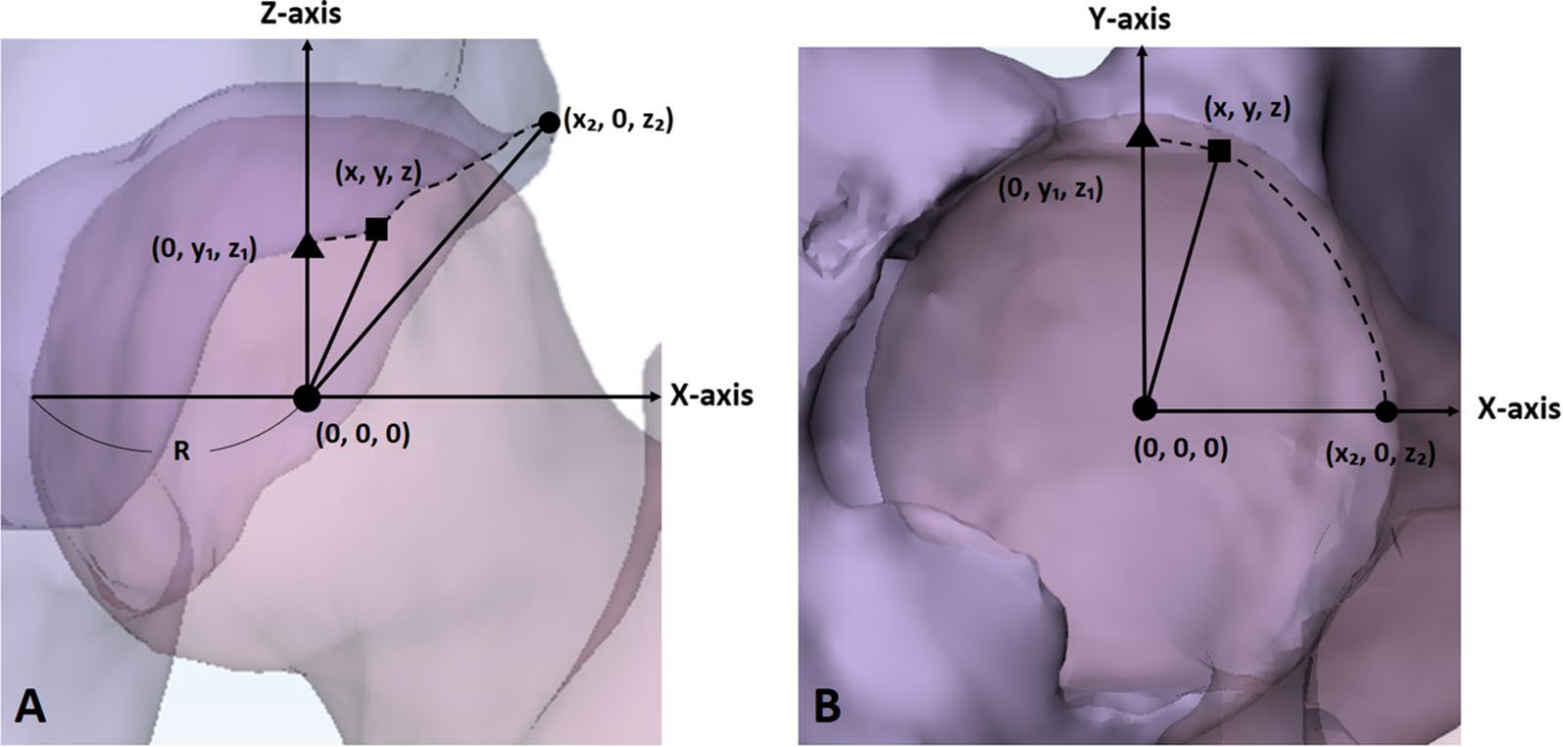
Example of the left hip. **A** Anterior view. **B** Inferior view. R: radius of the femoral head, ■: IP (x, y, z), ▲: MAP (0, y1, z1), ●: MLP (x2, 0, z2), dotted line: anterior–superior acetabular rim

To analyze the position of the anterior–superior acetabular rim, the coordinates of the IP, the most lateral point (MLP), and the most anterior point (MAP) of the rim based on the center of the femoral head were measured in the default posture. After setting the coordinates of the center of the femoral head to (0, 0, 0), the point where the acetabular rim passed through the YZ plane was defined as MAP (0, y1, z1), and the point where the acetabular rim passed the XZ plane was defined as MLP (× 2, 0, z2) (Fig. 4). To correct for size according to sex and individual, the relative distances on each axis were obtained by dividing the coordinate values by the radius of the femoral head. A larger distance along the X-, Y-, and Z-axes indicated that the IP was located in the lateral, anterior, and superior directions, respectively. Measurements of the acetabulum were performed on both sides. To evaluate intra- and inter-observer reliabilities, 3D models were remeasured 2 weeks after the initial measurements by two orthopedic surgeons (1st and 2nd author).

### Statistical analysis

A chi-squared test was used to compare AIIS ridge-type ratios between the sexes. An independent two-sample t test and a Mann–Whitney test were used to compare differences in age, coordinates, distance, radius, and acetabular coverage between the sexes. Effect sizes were calculated for differences between the sexes/AIIS ridge types using the standardized mean difference with 0.2, 0.5, and 0.8 considered as small, medium, and large effect sizes, respectively [5]. The intra- and inter-observer reliabilities of the measurements were assessed using intraclass correlation coefficients. Statistical analyses were performed using SPSS (version 25.0, IBM Inc., Armonk, NY, USA), and statistical significance was set at *p* < 0.05.

## Results

Mean ages were 38.87 ± 9.38 for men and 38.30 ± 9.59 for women, and there was no statistical difference (*p* = 0.803). The AIIS ridge type according to the location of the IP showed a statistically different ratio according to sex, and the proportion of the anterior type was higher in men (30.3%) than in women (7.6%) (*p* = 0.001, Table 1).

**Table 1:** AIIS ridge type according to sex

	Anterior type	Posterior type	Total
Men	30.3% (23)	69.7% (53)	100% (76)
Women	7.6% (5)	92.4% (61)	100% (66)

*AIIS* Anterior inferior iliac spine. *P* = 0.001 (chi-squared test)

Table 2 shows the comparison of measurements between the sexes. When comparing the sexes, the radius of the femoral head and acetabular coverage were statistically greater in men. IP coordinates for men were located anterior and inferior to those for women; there was no difference in X-axis coordinates. MAP coordinates for men were located inferior to those for women, while MLP coordinates for men were located lateral and inferior to those for women. When comparing AIIS ridge types, the radius of the femoral head and acetabular coverage were significantly greater in the anterior type. The IP coordinates of the anterior type were located medial, anterior, and inferior to those of the posterior type. The MAP coordinates for the anterior type were located inferior to those for the posterior type, while MLP coordinates for the anterior type were located lateral and inferior to those for the posterior type.

**Table 2:** Comparison of measurements between sexes

	Total	Comparison between sexes				Comparison between AIIS ridge types			
		Men (*n* = 76)	Women (*n* = 66)	*P* value	Effect size	Anterior (*n* = 28)	Posterior (*n* = 114)	*P* value	Effect size
Radius	21.60 ± 2.23	22.85 ± 1.91	20.20 ± 1.70	< 0.001	1.45	22.75 ± 1.96	21.34 ± 2.23	0.003	0.65
Coverage	0.83 ± 0.07	0.85 ± 0.07	0.82 ± 0.06	0.011	0.46	0.86 ± 0.06	0.83 ± 0.07	0.016	0.44
IP									
x/R	0.39 ± 0.17	0.39 ± 0.18	0.43 ± 0.18	0.223	0.22	0.24 ± 0.10	0.45 ± 0.17	< 0.001	1.32
y/R	0.80 ± 0.17	0.81 ± 0.18	0.73 ± 0.15	0.006	0.48	0.95 ± 0.09	0.73 ± 0.16	< 0.001	1.48
z/R	0.83 ± 0.16	0.77 ± 0.15	0.92 ± 0.14	< 0.001	1.03	0.68 ± 0.13	0.88 ± 0.15	< 0.001	1.37
MAP									
y1/R	0.97 ± 0.12	0.97 ± 0.11	0.98 ± 0.13	0.707	0.08	0.99 ± 0.09	0.97 ± 0.12	0.559	0.17
z1/R	0.64 ± 0.17	0.59 ± 0.17	0.69 ± 0.15	< 0.001	0.62	0.56 ± 0.12	0.66 ± 0.17	0.006	0.62
MLP									
×2/R	0.71 ± 0.12	0.75 ± 0.12	0.68 ± 0.11	0.001	0.61	0.78 ± 0.13	0.70 ± 0.11	0.001	0.70
z2/R	1.04 ± 0.14	0.98 ± 0.12	1.09 ± 0.17	< 0.001	0.76	0.97 ± 0.11	1.05 ± 0.16	0.013	0.53

All quantitative variables are presented as a mean ± standard deviation

*AIIS* Anterior inferior iliac spine, *IP* Inflection point, *MAP* Most anterior point, *MLP* Most lateral point, *d* distance from center of femoral head, *R* radius

The intra- and inter-observer reliabilities were as follows: 1.000/0.941 for the AIIS ridge type, 0.989 ~ 1.000/0.993 ~ 1.000 for coordinates of the IP, 1.000/1.000 for coordinates of MAP, and 1.000/1.000 for coordinates of MLP.

## Discussion

In this study, we found that the anatomical structure of the anterior–superior acetabular rim, indicating anterior focal coverage, differs according to sex and AIIS ridge type. These structural differences determine the location of the bony contact of the acetabular rim with the femoral neck and may also affect the development of FAI.

Although there are several methods to evaluate acetabular overcoverage for the diagnosis of pincer-type FAI [19, 30], it is not easy to accurately evaluate acetabular coverage with two-dimensional (2D) images because of the 3D structure of the acetabulum [18, 32]. In particular, since it is impossible to quantitatively evaluate anterior focal coverage of the retroverted acetabulum from 2D images, pincer-type FAI has been diagnosed using indirect methods, such as the crossover sign and posterior wall sign [25]. However, since these unreliable signs using a simple radiograph tend to overestimate acetabular retroversion [31], pincer-type FAI is reported to have a larger asymptomatic population than cam-type FAI [8]; therefore, compared to cam type, the relevance of pincer-type FAI to osteoarthritis is also considered relatively unclear [1, 15, 24]. To overcome this limitation, Dandachli et al. evaluated the validity of the crossover sign using a 3D CT model [7], and Murphy et al. suggested a method to quantitatively evaluate the crossover sign and focal acetabular overcoverage using a 3D CT model [22]. However, since the crossover sign compares relative positions of the anterior and posterior walls of the acetabulum, it is impossible to independently evaluate the anterior–superior acetabular rim (anterior wall) that actually affects FAI. Therefore, we analyzed the anatomical structure of the anterior–superior acetabular rim, which could not be confirmed in previous studies, by identifying the most prominent IP consistently found in the 3D models.

According to previous 3D simulation studies, osseous impingement of the hip joint occurs in the AIIS, AIIS ridge, and the acetabular rim around the ridge [2, 20]. Although bony contact does not occur only at the most protruding IP identified in our study, bony contact mainly occurs around the AIIS ridge in normal hips [20]. Because the IP of all acetabulums was located on the AIIS ridge, the IP in our study reflects the location of the surrounding acetabular rim, which indicates anterior focal coverage and the bony contact site of the anterior–superior acetabular rim.

**Fig. 5:**
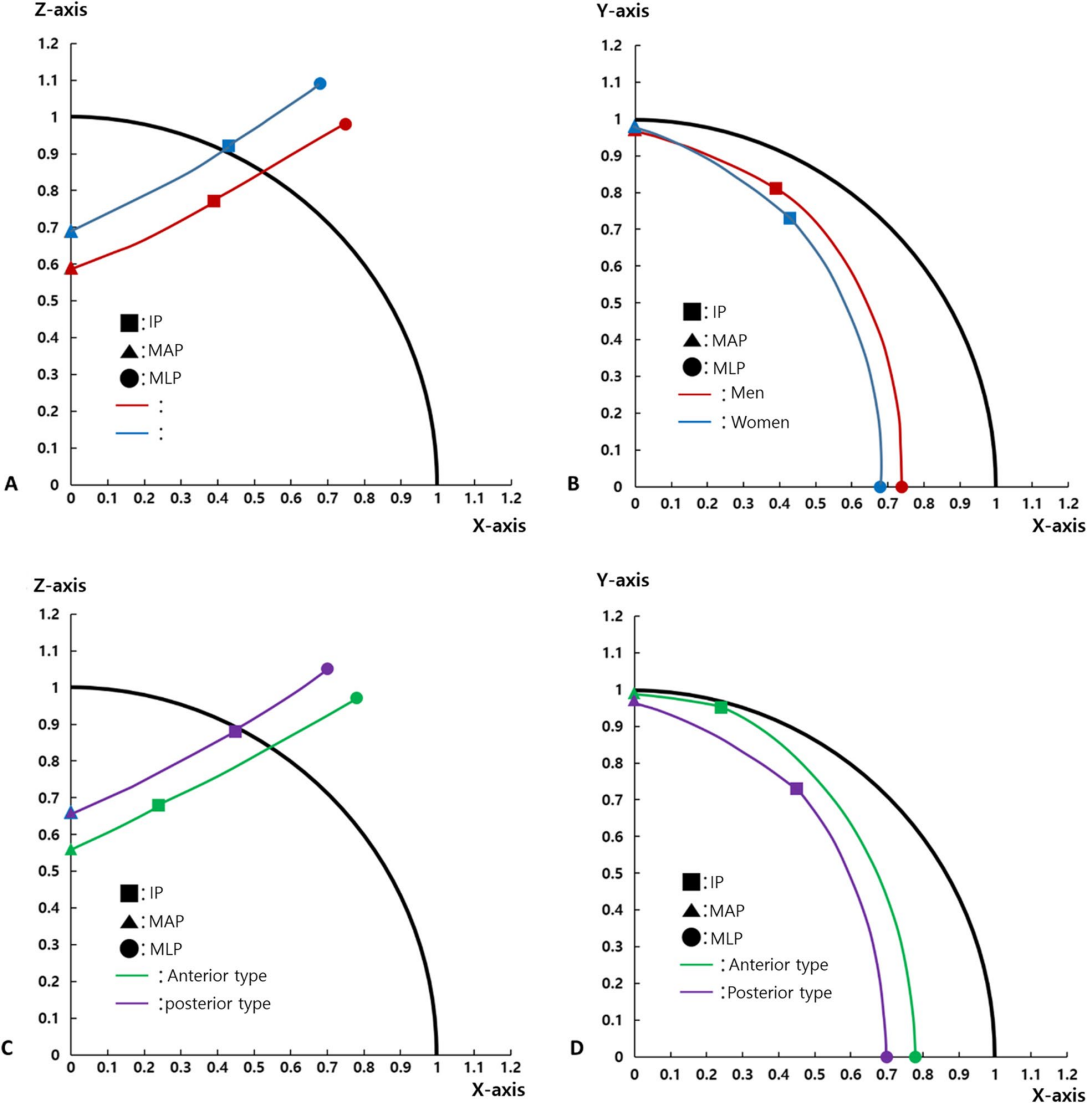
Schematic drawings of left anterior–superior acetabular rim. Comparison between sexes **A** from an anterior view and **B** from an inferior view. Comparison between AIIS ridge types **C** from an anterior view and **D** from an inferior view

As the pelvis and acetabulum show anatomical differences between sexes [23, 27–29], the structure of the anterior–superior acetabular rim also shows differences. Since the curved acetabular rim from the MAP to MLP in men is located anterolaterally to that in women in the XY plane and the coordinates along the Z-axis for the IP, MAP, and MLP are all smaller in men, the anterior focal coverage of the acetabular rim between the MAP and MLP including the IP was obviously greater in men (Fig. 5A, B). This indicates that bony contact occurs early during hip joint flexion and internal rotation, which is consistent with the results of previous studies in which men showed less hip joint ROM than did women [6, 12, 26]. In addition, since men show larger cam lesions in the femoral neck than do women [9, 10, 23] and the femoral neck of men tends to thicken with age [13], it increases the possibility of FAI with an increase in anterior–superior acetabular coverage. This supports previous research that reported cam- and pincer-type deformities to be more common in men [17, 21].

In our study, anterior focal coverage differed according to AIIS ridge types as well as sexes (Table 2). One of the reasons that men have greater anterior focal coverage than women appears to be that there are more anterior types in men (Table 1). Since the AIIS ridge of the anterior type (rim behind IP) is located anteriorly, inferiorly, and laterally, compared to that of the posterior type (rim anterior to IP), limitation of hip joint ROM and possibility of bony impingement are also greater (Fig. 5-C, D). As there are differences in ROM and the prevalence of FAI between sexes [6, 12, 17, 21, 26], who show differences in anterior focal coverage, it is expected that differences will also occur between AIIS ridge types. Therefore, an AIIS ridge of the anterior type could be a risk factor for FAI. Although this study was conducted as a cross-sectional observation study, further clinical studies are warranted to clarify disease patterns according to the anatomy of AIIS ridge.

This study had several limitations. First, because the patients were all Korean, different ethnicities may show different results. Contrary to our study, Tannenbaum et al. reported that anterior wall coverage was similar when comparing the acetabulum between sexes using CT scan in a mixed group of African American, Hispanic, and White [28]. Therefore, comparison of acetabular structures according to ethnicity could be another topic. Second, since it is a cross-sectional study targeting healthy people not related to hip disease, it could not provide a cutoff value for diagnosis and clinical results induced by anatomical differences. Therefore, a longitudinal study of the clinical results according to AIIS ridge type is necessary. Third, since the structure of the anterior–superior acetabular rim is 3D, it is difficult to identify it with simple radiographs or 2D CT images. Therefore, 3D reconstruction is required to be of use in actual clinical practice. However, this study is the first to classify type and to quantitatively evaluate the anterior–superior acetabular rim around the AIIS ridge, which is related to pincer-type FAI. Our results could help understand differences in the characteristics of hip disease between the sexes and contribute to suggesting treatment standards in the future.

## Conclusion

Anterior focal coverage of the acetabulum shows differences between sexes, and this difference may affect the development of pincer-type FAI. As between the sexes, anterior focal coverage also appears to differ according to the positioning of the AIIS ridge (anterior or posterior), suggesting that it may affect the development of FAI.

## Data Availability

The datasets generated during the current study are available from the corresponding author on reasonable request.
